# Changes in Patient Reported Pain Measures With the Citrate-free Adalimumab Formulation in Pediatric Inflammatory Bowel Disease Patients

**DOI:** 10.1097/PG9.0000000000000016

**Published:** 2020-10-21

**Authors:** Ashish S. Patel, Phuong Luu

**Affiliations:** From the * Division of Pediatric Gastroenterology, Phoenix Children’s Hospital, Phoenix, AZ; † Division of Pediatric Gastroenterology, Children’s Medical Center Dallas, Dallas, TX.

## Abstract

Inflammatory bowel diseases (IBD) are characterized by chronic inflammation in the gastrointestinal tract. The perception of pain in children is very complex and involves psychological, physiological, behavioral, and developmental factors. For children with chronic diseases, the medical procedures and treatments are often painful, unexpected, and heightened by situational stress and anxiety leading to an overall unpleasant experience. Pain and injection-site reaction are major predictors of nonadherence to antitumor necrosis factor treatment.

The most commonly reported adalimumab adverse event was injection-site reaction. This study compares reported pain in pediatric IBD patients between the 2 formulations using a visual analog scale (VAS). Our hypothesis is that the citrate-free formulation would have significantly less injection-site pain than the original formulation.

We evaluated injection-site pain in 6- to 17-year olds with IBD between the original formulation and citrate-free using the Faces Pain Scale-Revised for pain from 0 (no pain) to 10 (worse pain possible).

Ninety-five percent of patients reported that their pain score was greater than 3 with original formulation, while only 5% of them reported their pain score was greater than 3 with citrate free. The McNemar’s test showed significant difference in the pain score between the 2 types of injection (*P* < 0.0001).

**What is known**

- Antitumor necrosis factor biologic medications are one of the primary therapies in IBD in children.
- The perception of pain in children is multifactorial and has a direct effect on adherence.
- This is no published data on the comparison of pain between original and citrate-free adalimumab in pediatric IBD.

**What is new**

- The Faces Pain Scale-Revised VAS (FPS-R VAS) is an easy to use, reliable representation of a child’s pain perception.
- The FPS-R VAS scoring of injection-site pain was significantly (*P* < 0.0001) less in the citrate-free formulation when compared with the original formulation of adalimumab.

**I**nflammatory bowel diseases (IBD) such as ulcerative colitis and Crohn’s disease are characterized by chronic, remitting, and relapsing inflammation in the gastrointestinal tract. IBD in children is often associated with a more complicated and aggressive disease course. Unlike adults with IBD, improving and maintaining growth and pubertal development remain unique challenges in pediatric patients with IBD. The introduction and success of biologics, specifically antitumor necrosis factor (TNF) therapies in adult and children, has been a game-changer in the management of IBD. In pediatrics, the use of these biologics agents has increased the ability of the pediatric gastroenterologist to avoid corticosteroids in the highly susceptible growing child. This steroid-free remission is one of the primary goals in pediatric IBD.

Adalimumab (Humira), a fully humanized, anti-TNF alpha monoclonal antibody, was approved in 2002 for the treatment of multiple immune-mediated inflammatory diseases. Adalimumab (AbbVie, North Chicago, IL) is used worldwide for more than 10 indications like rheumatoid arthritis, juvenile idiopathic arthritis, psoriatic arthritis, ankylosing spondylitis, adult and pediatric Crohn’s disease, ulcerative colitis, plaque psoriasis, hidradenitis suppurativa, and uveitis (1).

In 2014, adalimumab received its FDA indication for the treatment of pediatric patients 6 years of age and older with moderately to severely active Crohn’s disease who have had an inadequate response to corticosteroids or immunomodulators such as azathioprine, 6-mercaptopurine, or methotrexate (1). The efficacy of adalimumab in inducing and maintaining remission in children with moderately to severely active CD was shown in the IMAGINE trial (2). The original adalimumab formulation was available in 40 mg/0.8 mL or 20 mg/0.4 mL subcutaneous autoinjector pen or syringe with a 0.27-gauge needle (1).

The perception of pain in children is very complex and involves psychological, physiological, behavioral, and developmental factors. Their perception of pain may also differ from adults due to developmental immaturity and differences in coping mechanisms. For pediatric patients with chronic diseases, like IBD, the medical procedures and treatments are often painful, unexpected, and heightened by situational stress and anxiety leading to an overall unpleasant experience. The recurrent painful experience related to injection-site pain, which is the most commonly reported adverse event for adalimumab, may adversely affect treatment adherence and compliance. Pain and injection-site reaction are major predictors of nonadherence to anti-TNF treatment (3).

Across all adalimumab indications, the most commonly reported adverse event was injection-site reaction, 20.3% compared with 13.8% in placebo (1). Pain associated with subcutaneous injection can be due to various factors, including inactive buffers, injection volume, and needle size (4–6). In response to these issues, a citrate-free formulation of adalimumab was introduced in May 2018. This formulation was specifically designed to reduce injection pain by removing the citrate acid monohydrate from the formula. The citrate-free adalimumab also contained 50% less injection volume (40 mg/0.4 mL compared with 40 mg/0.8 mL adalimumab formulation) while delivering the medication via a smaller gauge injection (29 vs 27 gauge) (1). The active ingredient of adalimumab remained unchanged in pharmacokinetic integrity and therapeutic efficacy. There is currently no published data evaluating injection-site pain between the original formulation and the new citrate-free adalimumab in pediatric patients with IBD. This study compares reported pain using a visual analogue scale (VAS) between the original citrate containing adalimumab formulation and the citrate-free adalimumab formulation in pediatric IBD patients. Our hypothesis is that the citrate-free formulation would have significantly less injection-site pain than the original formulation.

## STUDY DESIGNS/METHODS

Pediatric patients with IBD between the age 6–17 years that have used the citrate-free 40 mg/0.4 mL 29-gauge needle delivery and original adalimumab 40 mg/0.8 mL 27-gauge delivery formulation every 2 weeks were asked to rate their injection-site pain based on recall. We collected patient’s specific IBD type, gender, age, and VAS for pain. We evaluated changes in pain between the original formulation verses the citrate-free adalimumab using the Faces Pain Scale—Revised (FPS-R) for pain from zero (no pain) to 10 (worse pain possible). There was no randomization or crossover arm. The FPS-R VAS is commonly utilized in the pediatric setting and has been validated in pediatric pain assessment and adequately supported by psychometric data (7,8).

Injection-site pain is not disease specific and is the most commonly reported adverse event among adult and pediatric patients across all indications for adalimumab. Pediatric patients with ulcerative colitis or Crohn’s disease who were recently changed to citrate-free adalimumab from the original formulation were randomly selected to rate their perception of injection pain between the new and original adalimumab utilizing the FPS-R VAS for pain. All eligible patients were less than 18 years of age, had a confirmed histopathological diagnosis of IBD and had parental consent to participate. Patient selection for this study did not factor in disease activity at time of participation. The primary gastroenterologist or IBD nurse administered the FPS-R VAS pain scale during their routine office appointments. Subjects were asked to rate their injection-site pain using the VAS pain scale comparing the original verse citrate-free adalimumab.

The University of Texas Southwestern Medical Center IRB board approved the study.

## RESULTS

In 20 patients ranging from 11 to 18 years (12 male and 8 female; 16 Crohn’s disease; and 4 ulcerative colitis patients), 99% of patients reported injection pain with the original formulation 40 mg/0.8 mL adalimumab. The new citrate-free 40 mg/0.4 mL adalimumab formulation was associated with lower scores of overall injection-site pain. FPS-R VAS pain scale for original formulation adalimumab was 3–10 compared with 0–3 with citrate-free 40 mg/0.4 mL adalimumab. All the patients reported an improvement in injection-site pain with the citrate-free formulation. Nineteen out of 20 patients reported a significant decrease in injection pain for the citrate-free 40 mg/0.4 mL versus the original 40 mg/0.8 mL formulation and one patient reported minimal improvement.

Out of the 20 patients who received both citrate injection and citrate-free injection, 95% patients reported that their pain score (from 0 to 10 scale) were equal to greater than 3 when they received citrate injection, while only 5% of them reported their pain score were equal to greater than 3 when they received citrate-free injection. The McNemar’s test showed significant difference in the pain score between the 2 types of injection (*P* < 0.0001).

The reported improvement in injection pain was noted across all age group and disease type (ulcerative colitis or Crohn’s disease) (Tables 1 and 2).

## DISCUSSION

Subcutaneous injections are utilized in numerous medical procedures and treatments. Subcutaneous injections in children especially when recurrent often cause discomfort and pain that leads to decreased adherence or even cessation of treatment. Treatment adherence and injection reaction are even more important in children with chronic conditions that require long-term use of injections. The growth potential and window for these pre-pubertal and pubertal children is limited, and if growth is affected because of active disease or the use of corticosteroids, the negative effects are permanent. Once the window to grow is closed, meaning the growth plates are fused, there is no further potential to recover lost growth. Our study confirmed our hypothesis and demonstrated a significant improvement in injection-site pain with the citrate-free 40 mg/0.4 mL adalimumab formulation. In the future, a potential study to evaluate the effects of decreased injection-site pain on treatment adherence and patient experience on a larger sample size could further support our findings. One limitation of our study is that we did not capture specific data on location of injection site and that may further clarify or enhance our understanding of the injection experience. Understanding the factors related to injection-site pain and alleviating injection related pain are essential to assuring compliance and tolerability to chronic injectable medications like adalimumab.

## CONCLUSION

The new citrate-free 40 mg/0.4 mL adalimumab formulation was associated with significantly less injection site-related pain compared to the original 0.4mg/0.8 mL adalimumab formulation. The mean difference between the original and citrate-free adalimumab formulation on the patient-reported FPS-R VAS was significant. The citrate-free 40 mg/0.4 mL adalimumab provided an overall improved experience for pediatric IBD patients and their families. The tolerability of adalimumab, especially long term, is an important consideration in the pediatric population given the multiple indications approved for adalimumab and the desired long-term durability into adulthood.

Assuring compliance is paramount given the high costs associated with treatment. In the era of immunity-targeted therapies, antibody development may further limit the flexibility of switching classes. Noncompliance can result in antibody formation and subsequent loss of effect or potential dangerous drug reactions. Furthermore, the complications associated with sub-optimally controlled disease are more detrimental for children because the impact on their growth potential may be significantly affected. For those patients on weekly adalimumab dosing, the implications of compliance are even more important and improvement in experience with injection-site pain is even more impactful. Alleviating the burden of treatment may improve the quality of adherence and convenience for pediatric patients and thereby positively influencing treatment outcomes for patients with IBD. Besides efficacy, tolerability and safety, citrate-free adalimumab offers the advantages of convenience, portability, and autonomy that cumulatively promote treatment compliance. Reduced injection-site pain may contribute to enhance patient satisfaction with injectable therapy like adalimumab and help improve both patient’s adherence to therapy and long-term durability of the medication.

**Table 1.** Patient Demographics and Characteristics

Total (n)	20
Sex	
Male	12
Female	8
Age range (mean)	11–18 y/o (15)
IBD sub-type	
Ulcerative colitis	4
Crohn’s disease	16
Adalimumab dosing frequency	Every other week

IBD indicates inflammatory bowel diseases.

**Table 2.** Changes in Patient Perception of Pain on VAS by Category

VAS Category	Adalimumab Formulation n (%)	
	Original 40 mg/0.8 mL	Citrate-free 40 mg/0.4 mL
Mild (≤3)	1 (5%)	20 (100%)
Moderate (>3 to <7)	7 (35%)	0
Severe (≥7 cm)	12 (60%)	0

VAS indicates visual analog scale.
